# Broadening the Conception of Food Insecurity Among Older Adults: Development of a Summary Indicator in the NHATS

**DOI:** 10.1093/geroni/igaa057.2792

**Published:** 2020-12-16

**Authors:** Emma Tucher, Tamra Keeney, Alicia Cohen, Kali Thomas

**Affiliations:** 1 Brown University, Providence, Rhode Island, United States; 2 Providence VA Medical Center, Providence, Rhode Island, United States

## Abstract

Comprehensive measurement of food insecurity among older adults has focused on financial barriers to food access. Using data from the community-dwelling older adults in Round 5 of the U.S. National Health and Aging Trends Study (n=7,070), we constructed a summary indicator of food insecurity and inadequate food access incorporating items related to food scarcity within three domains: functional, social support, and financial limitations. We analyzed the construct validity of the summary indicator for known biopsychosocial factors associated with food insecurity among older adults. In 2015, 4.3% of community-dwelling older adults, approximately 1,673,775 million people, were characterized as food insecure or lacking adequate food access. Multivariable regression models identified that being homebound, frail, and experiencing community disability were associated with food insecurity and inadequate food access. These findings indicate food insecurity and inadequate food access among older adults is associated with functional and mobility characteristics and not isolated to financial barriers.

